# Comparison of Multiple NIR Spectrometers for Detecting Low-Concentration Nitrogen-Based Adulteration in Protein Powders

**DOI:** 10.3390/molecules29040781

**Published:** 2024-02-08

**Authors:** Matyas Lukacs, John-Lewis Zinia Zaukuu, George Bazar, Bernhard Pollner, Marietta Fodor, Zoltan Kovacs

**Affiliations:** 1 Department of Food Measurement and Process Control, Institute of Food Science and Technology, Hungarian University of Agriculture and Life Sciences, 1118 Budapest, Hungary; lukacs.matyas.krisztian@phd.uni-mate.hu; 2 Department of Food Science & Technology, Kwame Nkrumah University of Science & Technology, Kumasi-Ghana 00233, Ghana; zaukuu.jz@knust.edu.gh; 3 CORRELTECH Laboratory, ADEXGO Kft., 1222 Budapest, Hungary; bazar@agrilab.hu; 4 Independent Researcher, 6020 Innsbruck, Austria; bernhard.pollner@mac.com; 5 Department of Food and Analytical Chemistry, Institute of Food Science and Technology, Hungarian University of Agriculture and Life Sciences, 1118 Budapest, Hungary; fodor.marietta@uni-mate.hu

**Keywords:** near-infrared spectroscopy, chemometrics, food fraud, quality control, handheld NIRS, whey protein, melamine

## Abstract

Protein adulteration is a common fraud in the food industry due to the high price of protein sources and their limited availability. Total nitrogen determination is the standard analytical technique for quality control, which is incapable of distinguishing between protein nitrogen and nitrogen from non-protein sources. Three benchtops and one handheld near-infrared spectrometer (NIRS) with different signal processing techniques (grating, Fourier transform, and MEM—micro-electro-mechanical system) were compared with detect adulteration in protein powders at low concentration levels. Whey, beef, and pea protein powders were mixed with a different combination and concentration of high nitrogen content compounds—namely melamine, urea, taurine, and glycine—resulting in a total of 819 samples. NIRS, combined with chemometric tools and various spectral preprocessing techniques, was used to predict adulterant concentrations, while the limit of detection (LOD) and limit of quantification (LOQ) were also assessed to further evaluate instrument performance. Out of all devices and measurement methods compared, the most accurate predictive models were built based on the dataset acquired with a grating benchtop spectrophotometer, reaching R^2^P values of 0.96 and proximating the 0.1% LOD for melamine and urea. Results imply the possibility of using NIRS combined with chemometrics as a generalized quality control tool for protein powders.

## 1. Introduction

The human body requires protein to perform some important functions, which makes protein an important nutritional requirement. There has been a recent surge in the demand for its semi-processed forms, mostly as dietary supplements, in urban areas, due to the fast-tracked lifestyle of the majority of the urban population [1]. The recommended dietary reference intake of protein per kilogram of body weight is 0.8 g [2], and this is equivalent to 56 g per day for adults who do not undertake any daily activities that expend high amounts of energy [1]. Considering individuals who indulge in extensive and/or intensive workouts and exercises, there is a higher demand for protein to compensate for the extra energy expenditure and to provide the building blocks for muscle growth.

The study by Andrade et al. (2019) [3] reported that the consumption of protein-rich nutritional supplements has been increasing over the years to compensate for the increase in protein needs. A popular example of a protein supplement is whey protein (WP) powder. Whey protein, a common by-product of the cheese industry, is a highly bio-available protein source rich in essential amino acids with proven benefits for post-exercise muscle synthesis [4,5], making it a popular choice among consumers partaking in physical activities. The allergenic compound-related limitations of whey protein and an increase in specialized diets, including veganism [6], have led to the emergence of a wide variety of additional protein powders on the market, including pea and beef protein. In ensuring the quality of protein-based foods, such as protein powders, several analytical methods have been explored. The most common among these are based either on combustion (Dumas) or chemical digestion (Kjeldahl), techniques capable of assessing protein concentrations in food products based on their total nitrogen (N) content. In these methods, the Kjeldahl conversion factor of 6.25 is most commonly used to express protein content from the measured total nitrogen [7], but it has some issues of accuracy. The calculation of the conversion factor is based on the amino acid profile of the protein, which, depending on the type of protein, can vary. An initiative by the Codex Alimentarius committee published that a standard conversion factor of 6.38 rather than 6.25 should be used to determine protein in milk products [7]. Still, these methods evaluate the so-called apparent protein content based on the sample’s total nitrogen content, not specifically nitrogen derived from proteins.

Zhang and Xue (2016) [8] report an increase in adulteration of the total nitrogen content of food products such as raw milk, cereal-based formulations, and powdered infant formula with the addition of melamine (C_3_H_6_N_6_) in many countries, including China. Melamine is an organic nitrogenous compound synthesized from urea and is used in the production of plastics, dyes, fertilizers, and fabrics. The primary reason behind the use of this compound as an adulterant is due to its high (66.6%) nitrogen content, which makes it an “ideal” tool to increase the pseudo-protein content of certain food products. The addition of this nitrogenous compound to protein-based food products may be harmful, as melamine consumption has been linked to nephrolithiasis, chronic kidney inflammation, and bladder carcinoma [9]. Urea (CH_4_N_2_O) is a well-known non-protein nitrogen source in the feed market, widely used to supplement the diet of cattle or other ruminants, as ruminal urea is rapidly hydrolyzed by the bacterial urease enzyme into carbon dioxide and ammonia, which are dominantly used during bacterial protein synthesis [10]. On the contrary, monogastics (e.g., poultry, pigs, and humans) can digest only real proteins and use only them in their bodies; therefore, large concentrations of urea may cause health problems [11]. Due to its presence in the feed market in general, it is sometimes used as an adulterant in high-quality protein meals, such as gluten [12]. While using amino acids as additives to protein-rich food commodities might not raise similar health-related risks as urea or melamine adulteration, it still introduces a form of consumer deception when calculated based on the protein content of these products. The procedure of using cheap amino acids to modify the product’s nitrogen content is called amino acid-spiking, which to this day is a rather uncharted field among available scientific studies, despite its frequent occurrence in industry [13].

Within the food industry, the authenticity and authentication of products are emerging topics [14], in which ensuring ingredient quality throughout the entire production process requires a holistic approach. Total nitrogen determination methods are incapable of reliably assessing a sample’s protein content in the case of nitrogen-based adulteration, while they are also subject to high consumable and time costs and bound to a laboratory environment [13]. Therefore, affordable alternative approaches with better sensitivities are required. An example of a non-sophisticated technique that can serve as an alternative approach to the total nitrogen determination methods is near infrared spectroscopy (NIRS). Technically, near-infrared instruments operate on the Beer–Lambert principle of light with a wavelength range between 800 and 2500 nm, but the spectral intervals and other instrumental characteristics may vary depending on the producer [15]. Generally, the technique measures the intensities of absorptions of radiation while NIR light passes through a sample. There are various kinds of technological solutions to acquire the NIR absorption spectra. For example, when the infrared light from the light source passes through a Michelson interferometer along the optical path, it is called Fourier transform infrared spectroscopy (FTIR) [16]. FTIR spectroscopy is based on interferometry and makes use of the complete source spectrum rather than recording the spectra at the individual wavelengths that can be generated by grating or prism systems used in conventional near-infrared spectroscopy [17]. DLP-based spectrometers include a digital micromirror device (DMD) and a single-point detector for wavelength selection, making them more suitable for portable designs than spectrometers with conventional linear array detectors [18].

NIR spectrometers are no longer limited to laboratory-bench-compatible instrumentations. In recent times, there have been several developments in portable and hand-held forms of NIR spectrometers. These spectrometers, which are miniature versions of vibrational spectroscopy equipment, have made it possible for the food industry to do on-site and real-time evaluations of food quality and food production processes. They have been used for authentication and detection of adulterants in several foods [19,20,21], food product development [22,23], and geographical discrimination of food [24,25,26].

Recent debates about instrumental efficacy have led to more comparisons between benchtop and handheld instruments using advanced mathematical techniques known as chemometrics. These techniques help to access the performance of NIR instruments through exploratory data analysis [27], classification [28], and prediction [29]. Through these techniques, parameters of quality control such as sensitivity, specificity, precision, detection and quantification limits, and various predictive modeling parameters can be accessed. The term “calibration transfer” is a rapidly emerging topic in the NIR community [30,31,32], supposed to provide ways to build robust databases and models using one NIRS device (usually benchtop), while leveraging these models with other devices to make predictions without building entirely new databases and models. Whereas the process could significantly reduce the most time-consuming and costly part of NIRS applications, successfully transferring calibrations between devices requires a thorough understanding of the instrument setup, performance, and available chemometric tools.

In two recent studies [1,13], protein powder adulteration with multiple potent nitrogen-based adulterants, including amino acids, was reportedly detected using near-infrared spectroscopy, but comparisons between instruments and their performance were limited. Also, both studies only focused on whey protein powder, whereas several other types of protein powders are present on the market to supply various dietary needs. Zaukuu et al. (2020) [1] also found it feasible to build NIRS models by acquiring spectra through packaging (LDPE bag), an important aspect for applying NIRS as a monitoring tool for quality control during the whole production.

Based on scientific literature, to the best of our knowledge, no study has reported a comparison of multiple NIR instruments for the detection of very low concentrations of various adulterants in whey, beef, and pea protein powders. To bridge this gap, our study aims to assess the performance of three benchtop and one handheld NIR instruments with two different sample presentation methods for the rapid detection and quantification of multiple adulterants in whey, beef, and pea protein powders. The aim was also to prepare a solid groundwork for future developments, including the building of more generalized and potentially transferrable predictive models to support the quality control of multiple types of protein powders.

## 2. Results

### 2.1. Exploratory Data Evaluation of the NIR Spectroscopy Results Based on Raw Spectra

**Figure 1:**
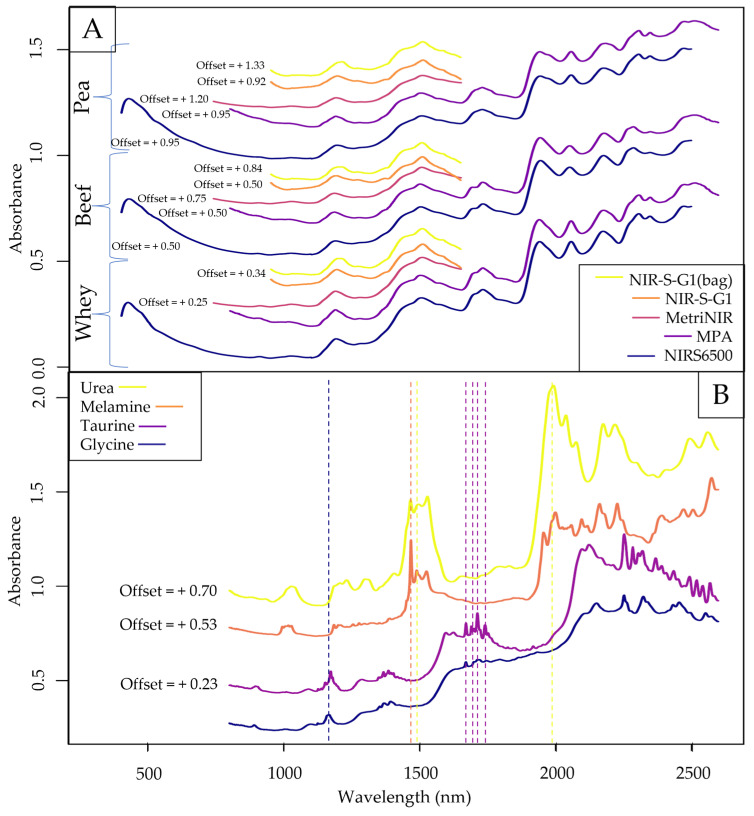
Average raw spectra of pure protein powders (**A**) and each pure adulterant recorded with the FT-NIR (MPA) instrument (**B**). Vertical markers refer to some of the more significant characteristic absorbance peaks of a given adulterant.

FT-NIR (MPA) was chosen for exploratory visualizations because it had the narrowest bandpass among the involved spectrometers. The averaged raw spectra of each control set (pure protein powders) and pure adulterants can be seen in Figure 1. Based on Figure 1A, a sizeable baseline shift was visible for both handheld datasets, most notably for the spectra acquired through the optical glass (NIR-S-G1). The primary reason behind this observation is that the position of the light source and sensor of the NIR-S-G1 is calibrated for contact measurements, where any distance between the device and the measured sample might change the angle in which the reflected light is recorded, introducing a systematic shift in absorbance values. Using a glass window cuvette for spectral acquisition induces a large enough gap for this phenomenon to be observed, whereas the thin-layer LDPE bag mimics contact measurements more closely. For this reason, spectral pretreatment for baseline-shift correction was always included in the case of handheld data for further evaluations.

From observations in Figure 1A, the only separable sub-datasets were the ones recorded with the MetriNIR, where the absorbance values in the lower and higher wavelength ranges followed a different pattern than that of the other devices. By visual interpretation, there was no noticeable difference between the spectra of different protein types, suggesting the possibility of including all protein types in the same dataset for modeling. This statement was later evaluated by PCA results.

Observing the raw spectra of the pure adulterants (Figure 1B), a very distinct peak for melamine can be seen at ~1480 nm, close to the prominent peaks of urea at ~1490 and ~1530 nm. Since these compounds contain a significant amount of nitrogen, the expression of absorbance peaks in the 1st overtone region with N-H stretches (~1400–1600 nm) is expected. Taurine, the only sulfur-containing adulterant in this study, has multiple prominent peaks in the ~1650–1750 nm range, which falls under the S-H stretches of the 1st overtone region. Based on the raw spectra, glycine has the least distinct absorbance peaks, only sharing a prominent peak with taurine in the 2nd overtone region around 1200 nm.

**Figure 2:**
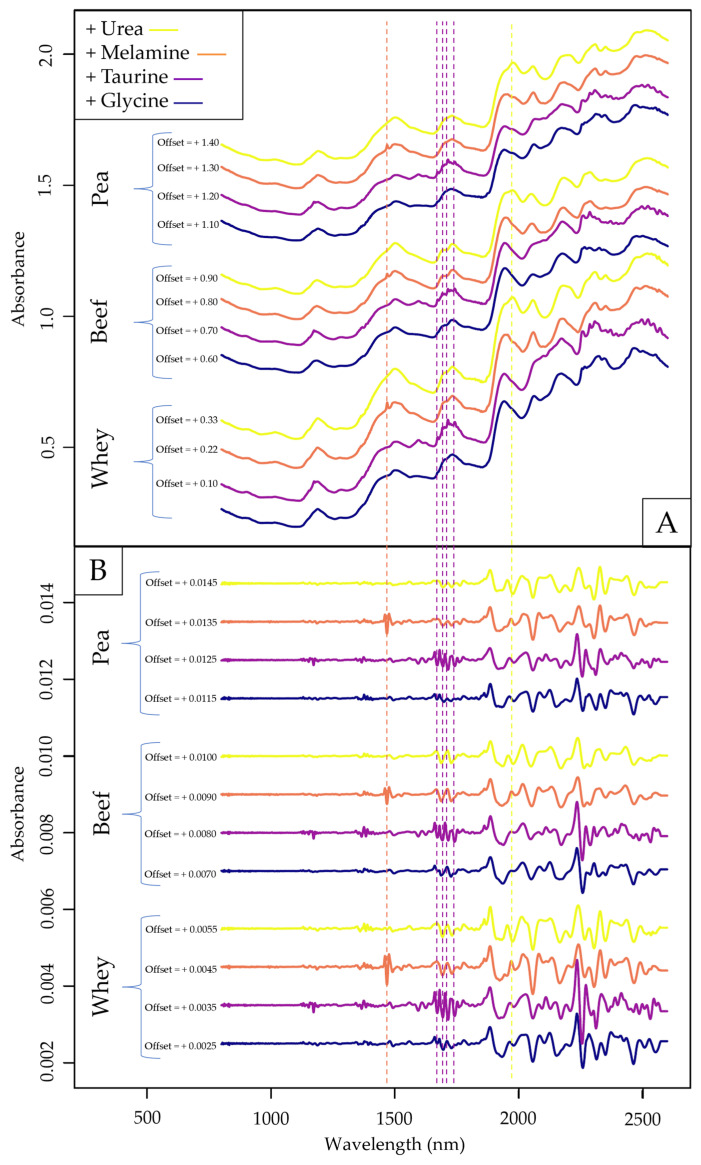
Average raw spectra of samples with the highest singular adulterant concentration (**A**) and their second derivatives (**B**) recorded with the FT-NIR (MPA) device. Vertical markers indicate previously identified prominent absorbance peaks visible in either of the panels.

The averaged raw spectra of samples with the highest single adulterant concentration can be seen in Figure 2A, and their second derivatives in Figure 2B. Some of the previously identified absorbance peaks (Figure 1) are also visible in Figure 2, most notably the melamine peak at ~1480 nm, which can be clearly identified in both panels of the figure. Taurine also has distinguishable peaks in the 1st overtone region with S-H stretches similarly to the observations in Figure 1, which might be because taurine has a much higher sulfur content than whole protein sources. The more prominent urea peaks at 1490 and 1530 nm (Figure 1) are not visible when the compound is mixed with protein powders; only the peak at ~1980 nm can be clearly identified. As for glycine, there are no distinguishable absorbance peaks in Figure 1 by visual inspection.

**Figure 3:**
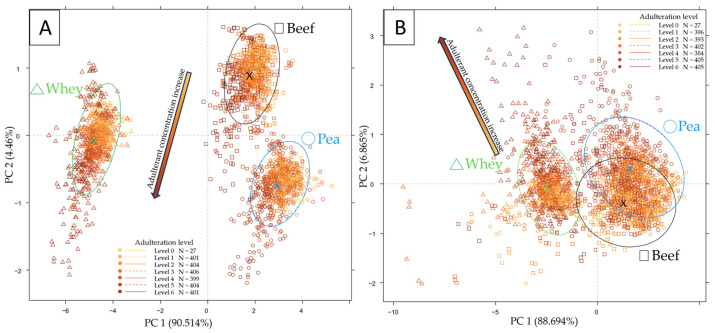
PCA score plot for the MPA dataset (**A**) and the NIR-S-G1 (bag) (**B**) dataset, using the entire available spectral region for both devices. Savitzky–Golay filter with 2nd-order polynomial and 21 smoothing points and SNV were applied as pretreatments.

Upon observing the raw spectra in Figure 1 and Figure 2, the presumably best-performing benchtop (MPA) and handheld (NIR-S-G1 with plastic bag) datasets were visualized with PCA after applying the necessary spectral pretreatments (Figure 3). The Savitzky–Golay with a 2nd-order polynomial and 21 smoothing points in combination with SNV has proven to be the overall most suitable for reducing spectral noise and scattering effects that were visible on the raw spectra. The previous implication that the different protein types might not show significant differences can be rejected based on the PCA results, as the whey, beef, and pea datasets are clearly separable, especially with the benchtop dataset (Figure 3A). The PCA measurement points on the score plots also show an incremental tendency based on adulteration levels alongside PC2 in both cases, while the measurement points belonging to different protein types separate alongside PC1. In the case of the benchtop instrument (Figure 1A), the average distance to the cluster center is lower, while the distance to other centers is noticeably higher than in the case of the handheld device (Figure 1B), resulting in less spread and more measurement points in the confidence interval. Another thing to notice is that the spread of the measurement points seems to be the lowest for whey protein samples in both cases.

After the exploratory data evaluation based on raw spectra and PCA, each dataset was split into three sub-datasets (whey, beef, and pea) for further evaluation, while restricted wavelength ranges were selected for each instrument (NIRS6500: 1100–2200 nm; MPA: 1400–2200 nm; MetriNIR and NIR-S-G1: 950–1650 nm) to focus only on the regions with the most distinguishable peaks. The wavelength regions above 2200 nm were ignored due to their future applicability, as measurements with fiber optic probes induce a high amount of noise in the higher wavelength spectral regions, according to previous experience and literature [33].

### 2.2. Supervised Classification of the NIR Spectroscopy Results Based on LDA

**Figure 4:**
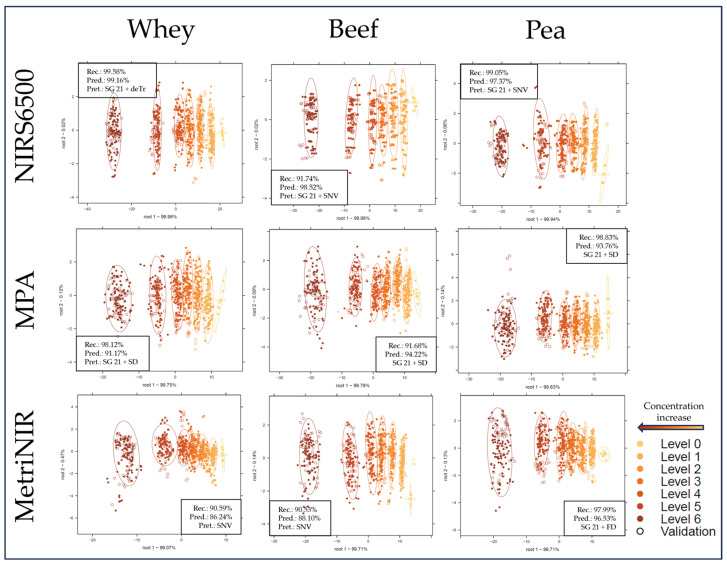
LDA classification score plots with confidence intervals (circles) and average accuracy results for benchtop sub-datasets. “Rec.” = average correct recognition of the calibration; “Pred.” = average correct prediction of the cross-validated model; “Pret.” = applied pretreatments; “SG 21” = Savitzky–Golay filter with 21 smoothing points.

**Figure 5:**
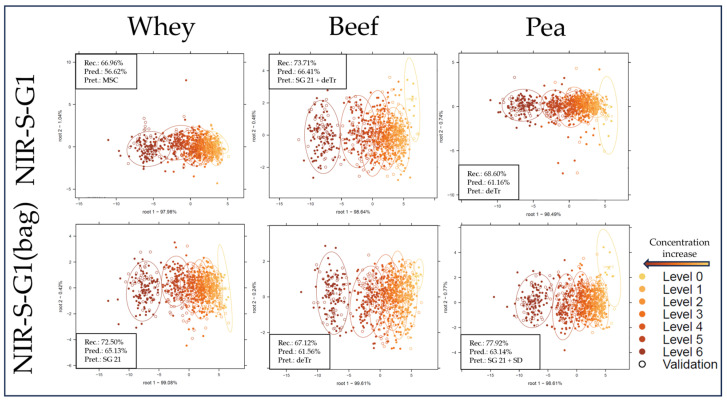
LDA classification score plots with confidence intervals (circles) and average accuracy results for handheld sub-datasets. “Rec.” = average correct recognition of the calibration; “Pred.” = average correct prediction of the cross-validated model; “Pret.” = applied pretreatments; “SG 21” = Savitzky–Golay filter with 21 smoothing points.

Figure 4 and Figure 5 show the LDA classification results based on each sub-dataset to separate the different adulteration levels. All LDA score plots show a similar tendency, where measurement points separate alongside the first discriminant function from right to left, with more overlap between groups for the handheld datasets (Figure 5). Average prediction accuracies were significantly (F(1, 43) = 274.2, *p* < 0.001) higher for benchtop devices compared with handheld devices. While comparing the performance based on different protein types, the models based on pea protein sub-datasets provided the overall highest, while the whey sub-datasets provided the overall lowest average correct classification accuracies, although the differences were not significant (F(2, 42) = 0.11, *p* = 0.9). As for instrument performance comparisons, models based on NIRS6500 data show the highest cross-validated correct classification accuracy, with 99.16%, 98.52%, and 97.37% for the whey, beef, and pea protein sub-datasets, respectively, significantly (F(1, 16) = 8.92, *p* < 0.01) higher values than in the case of the FT-NIR (MPA). A previous study using a similar FT-NIR device to classify melamine adulteration in milk powder reported increasing misclassification at lower (≤0.4% *w*/*w*) concentration levels [34]. Other authors could reach similar classification results using an FT-NIR device [35]. In agreement with observations during exploratory data analysis, although not significantly (F(1, 16) = 0.44, *p* < 0.01), the models based on the NIR-S-G1 dataset acquired by scanning through the plastic bag reached overall higher predictive accuracy than its glass-cuvette counterpart, with the exception of the models built on the beef protein sub-dataset. The observation that scanning through a plastic bag compared with a glass cuvette shows slightly better results is in agreement with previous findings [1].

### 2.3. Building Prediction Models Based on NIR Spectroscopy Results with PLSR

PLSR modeling results based on each sub-dataset to predict adulterant concentrations, as well as protein powder content, can be seen in Table 1, Table 2, Table 3, Table 4 and Table 5. To predict protein powder content (Table 1), the models built with NIRS6500 data reached the highest cross-validated R^2^ values of 0.96 and 0.95 for the whey and beef sub-datasets, respectively, with a corresponding average error of 0.71 and 0.79 g/100 g. The model built with MPA based on the pea sub-dataset provided the best parameters, with an R^2^CV value of 0.95 and an RMSECV value of 0.79 g/100 g. Comparing the handheld models, for protein powder content prediction, the models based on the glass-cuvette dataset provided the more accurate predictive results, with R^2^CV values of 0.81, 0.88, and 0.89 and respective RMSECV values of 1.58, 1.25, and 1.22 g/100 g for the whey, beef, and pea sub-datasets.

As for the prediction of urea concentration (Table 2), models based on NIRS6500 data were the most accurate for the whey and beef protein matrices, with respective RMSECV values of 0.19 and 0.18 g/100 g and an R^2^CV value of 0.95 in both cases. These predictive accuracies are comparable to previous research using Raman spectroscopy to detect low-level urea adulteration [36], where the authors could reach >90% accuracies with PLSR-based modeling in the 50–100 mg/100 mL concentration range. The lowest LOD and LOQ values were reached with the NIRS6500 PLSR models in all three cases, with LODmin, LODmax, LOQmin, and LOQmax values as low as 0.04, 0.13, 0.13, and 0.39 g/100 g for the beef sub-dataset. Comparing handheld model performances, the model based on the dataset recorded through a plastic bag reached notably better model parameters to predict urea concentration in whey protein, with an R^2^CV of 0.92 and an RMSECV of 0.24 g/100 g. For the prediction of urea in beef and pea protein, models built with both handheld datasets had similar results.

For the prediction of melamine concentration (Table 3), model performances were much more diverse. By focusing on the whey protein sub-dataset, the NIRS6500 provided the best model parameters with an R^2^CV of 0.94 and RMSECV of 0.15 g/100 g, whereas the model based on the handheld glass-cuvette dataset, aside from LOD and LOQ values, had better parameters than its plastic bag counterpart. The same observation can be made for the handheld models when focusing on the beef protein sub-datasets, while in the case of pea protein, the model based on plastic bag measurements decisively performs better. Returning to benchtop comparisons, models based on data acquired with the MetriNIR had the overall best performance in the case of beef and pea protein, except for the LOD and LOQ values, where lower values are achieved with models based on NIRS6500 and MPA results. In a previous study by Mauer et al. (2009) [37], the authors built highly accurate (R^2^CV > 0.99) PLSR models to predict melamine concentration in infant formulas in the 0.0001–50% (*w*/*w*) concentration range. Although the lowest concentration level (1 ppm) was successfully separated from unadulterated samples with a confidence level of 99.99% and a selectivity >2 using factorization analysis, an RMSECV value of 0.62% (*w*/*w*) was achieved for the PLSR model, presumably due to the wide concentration range applied, whereas the limit of detection values were not calculated. In the present study, out of all the 75 created models, the NIRS6500 has the lowest LODmin, LODmax, LOQmin, and LOQmax values of 0.03, 0.12, 0.10, and 0.37 g/100 g when focusing on the beef sub-dataset. This limit of detection goes beyond the lowest melamine concentration in the dataset, which is 0.13 g/100 g (UGTM; level 1).

While predicting taurine concentration (Table 4), the model with the best performance metrics was built based on the NIRS6500 whey protein sub-dataset, with an R^2^CV of 0.96 and RMSECV of 0.40 g/100 g. When focusing only on beef and pea protein, however, the models built with MPA data had decisively the best predictive performances, including LODmin, LODmax, LOQmin, and LOQmax values as low as 0.06, 0.15, 0.18, and 0.45 g/100 g. These values are a magnitude lower than those reported by previous research focusing on amino acid-based adulteration [38]. Comparing the handheld models, the ones based on the plastic bag dataset had the overall better performance when predicting taurine concentration in whey and beef protein powders, whereas the model based on measurements with a glass cuvette had notably better performance in the case of pea protein with an R^2^CV of 0.89 and RMSECV of 1.19 g/100 g.

Table 5 summarizes the PLSR models predicting glycine concentrations. By far the best predictive performances were achieved with the models based on NIRS 6500 data in the case of all the protein powders. The best model was built while focusing on whey protein with an R^2^CV of 0.96 and RMSECV of 0.68 g/100 g, while slightly better LODmin, LODmax, LOQmin, and LOQmax values of 0.04, 0.14, 0.11, and 0.41 g/100 g were achieved by focusing on the beef sub-dataset. As for the handheld comparison, in the case of whey protein, the model based on data recorded through the plastic bag had notably better performance metrics, while for the beef and pea sub-datasets, it was the other way around.

As also indicated by the comparisons made to previous studies above [30], RMSE and LOD/LOQ values highly depend on the concentration range of the detectable analyte. This is one of the explanations for the variance in LOD/LOQ and error values across different adulterants, as the present study aimed at modeling real industrial adulteration scenarios with the selected concentrations rather than reaching the lowest possible detection limits. For the latter, concentration ranges approximating the presumable detection limit should be used, while avoiding bias toward the accurate prediction of samples with high analyte content using wide concertation ranges, caused by the minimization of mean squared error (MSE) in many multivariate algorithms, as described in literature [39,40].

Combining all previous results, for further evaluation, the NIRS6500 and the NIR-S-G1 (plastic bag) datasets were chosen.

**Figure 6:**
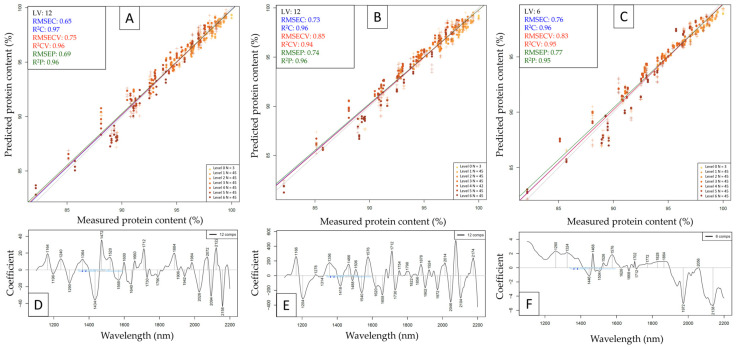
Test-set validation to predict protein powder content based on data collected with the NIRS6500 instrument. **A** = Y-fit plot with whey protein as sub-dataset, **B** = Y-fit plot with beef protein as sub-dataset, and **C** = Y-fit plot with pea protein as sub-dataset; “**D**”, “**E**” and “**F**” refer to respective regression vectors.

**Figure 7:**
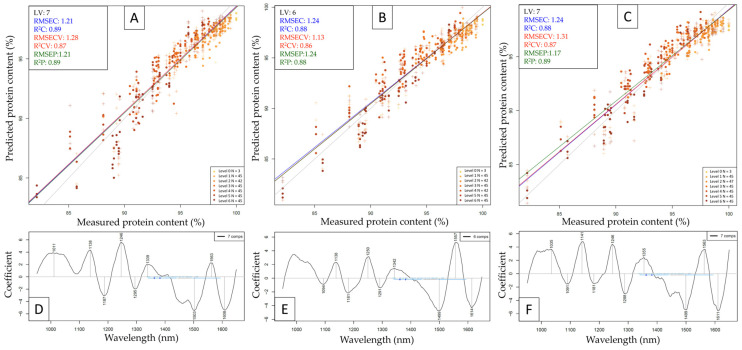
Test-set validation to predict protein powder content based on data collected with the NIR-S-G1 handheld instrument while scanning through a plastic bag. **A** = Y-fit plot with whey protein as sub-dataset, **B** = Y-fit plot with beef protein as sub-dataset, and **C** = Y-fit plot with pea protein as sub-dataset; “**D**”, “**E**” and “**F**” refer to respective regression vectors.

The results of test-set validation to predict protein powder content in the presence of adulterants based on the NIRS6500 dataset can be seen in Figure 6, while the results based on the NIR-S-G1 (plastic bag) dataset can be seen in Figure 7. The test-set validated NIRS6500 models had comparably high performances, where the model built to predict whey protein content proved to be the most accurate with an R^2^P value of 0.96 and an RMSEP value of 0.69 g/100 g. This is comparable to previous results using the MPA FT-NIR [13] with an R^2^P value of 0.99 and an RMSEP of 0.45% (*w*/*w*) to predict protein content in adulterated whey protein samples, although the study used a less complex matrix with fewer adulterants simultaneously. Several major absorbance peaks become visible on all three regression vectors in the 1st overtone region with N-H stretches (~1400–1600 nm) and in the region of combination bands with N-H stretches (above ~2000 nm. There are also a few shared peaks in these regions among different protein powder types by looking at the regression vectors (Figure 6D–F); for example, whey and beef share prominent peaks at ~1166, ~1660, and ~1712 nm, whereas major peaks are visible for beef and pea at ~1466, ~1576, and ~1972 nm. Regression vectors based on whey and pea datasets do not have major common absorbance peaks, which aligns well with the observations on the PCA score plots (Figure 3), where measurement points of pea and whey samples are positioned the furthest from each other. 

Out of the three test-set validated models based on handheld data, the model predicting pea protein content had slightly better performance metrics than the other two (Figure 7), with an R^2^P value of 0.89 and an RMSEP value of 1.17 g/100 g. By observing the regression vectors (Figure 7D–F), almost all the major peaks are shared among multiple protein types, most notably at ~1138, ~1181, ~1246, ~1499, and ~1563 nm, whereas protein-specific absorbance peaks are scarce.

## 3. Discussion

As near-infrared spectroscopy relies on the absorption properties of samples without being exclusive to any singular chemical element, like nitrogen, it can be a suitable tool to identify foreign substances in protein-rich food matrices, as proven in present and previous studies [1,13,40]. Previous research explored the applicability of NIRS combined with chemometric tools to assess the real protein content of whey protein powder in the case of simultaneous adulteration with multiple compounds [13], reaching similar predictive model performance and LOD/LOQ values to the present study. Additionally, the performance of a benchtop and a handheld instrument was compared previously to detect nitrogen-based adulteration in whey protein powder, reaching comparable predictive accuracies with RMSEP values ranging between 0.21 and 1.14 g/100 g for the benchtop device [1].

The present study builds upon the previous findings and adds several additional contributions. Multiple NIR spectrometers with different spectra-processing techniques were compared, out of which the NIRS6500 with grating technology provided the most accurate models overall, outperforming the FT and DLP spectrometers in almost all cases. Whereas multiple studies have reported highly accurate predictive accuracy using FT-NIR (mainly MPA) data for model building [13,34,35,37,40], for the present sample set and modeling combination, the NIRS6500 has proven to be more suitable in most scenarios. As many of the previous publications used non-linear algorithms for modeling, it would be reasonable to revisit the datasets by comparing the performance of multiple algorithms. Based on previous findings [1], the inferior performance of the handheld device was expected, but the models built with the NIR-S-G1 had comparable predictive accuracy to the benchtop device. An interesting finding is that the handheld device overall performed better when scans were collected through an LDPE bag instead of a glass cuvette. This systematic shift in absorbance values (also visible by observing the raw spectra) could contribute to the larger distance between the light source and the reflective surface in the case of using a cuvette, as the light source-sensor pair is calibrated for contact measurements for the DLP device. This piece of information is vital for future developments regarding the instrument, as it encourages applications that mimic contact measurements as closely as possible. Since NIR technology was proven suitable for miniaturization and such devices could potentially enable on-sight/in-line applications, which is desirable in many fields of the food industry, the authors believe that research and development should continue side-by-side to extend the use of these devices. A recent study by Bec et al. (2020) [41] expressed a concern that the development of miniaturized devices still requires comprehensive research and validation in laboratory environments, which is why it is suggestible to include them in comparison studies like the present one. Moreover, as NIR technology becomes more widespread in industrial applications and so does the need for generalized and transferrable models, portable instruments leveraging models built in laboratory environments could become an ever more important research topic.

LOD and LOQ values were thoroughly calculated for a large number of models, and the NIRS6500 spectrometer could reach the lowest values, approximating the 0.1% limit in the case of urea and melamine detection. Balabin et al. (2011) [27] could reach sub-ppm LOD values for melamine in milk products by combining NIRS with non-linear machine learning tools, including support vector regression and artificial neural networks, implying the possibility to build models with extremely high sensitivity given a suitable dataset and the right algorithm. The authors identified non-linear patterns between NIR spectral data and complex pseudo-protein (melamine) concentrations while investigating milk products, encouraging the use of algorithms that can bypass the issues of non-linearity. This was also implied based on the finding of Zhang et al. (2014) [35], where the authors were focusing on optimizing support vector machine and K nearest neighbors-based models to accurately classify pseudo-proteins (notably melamine and urea) in very low (0.04–0.11% and 0.05–0.16%) concentrations, although while investigating different matrices (raw milk). Regarding datasets, the present study investigated not just one but multiple protein powder matrices as targets of nitrogen-based adulteration. While the separation of measurement points on PCA score plots implies otherwise, observations on raw spectra and PLSR regression vectors suggest the possibility of treating samples belonging to different protein powder types as a single dataset. One of the biggest downsides of using PLSR for prediction is that PLSR models are sensitive to variations in the underlying data distribution, and when having significantly different matrices, the relationship between the spectral data and the concentrations of the adulterants may largely vary [42]. Therefore, the investigation of merging datasets belonging to different matrices and applying non-linear predictive algorithms for data evaluation is proposed as a future development with the aim of supporting a more generalized application of NIRS in the quality control of protein powders.

An additional proposal is the investigation of calibration transfer as a way of reducing the work required to apply different NIR instruments in quality control scenarios for these products. The present study has compared the performance of multiple NIR spectrometers by designating two device/measurement method combinations (NIRS6500 as benchtop and NIR-S-G1 with a plastic bag as handheld), where transferring calibrations could be worth investigating in future research. The achieved results may help the wider application of NIR technology in the quality assessment of protein sources. This implication would be important not only for the food industry and human health but also for the feed industry and animal health, as the mentioned protein adulterations are causing significant losses in animal farming due to resulting gain losses or even lethal disorders [43].

## 4. Materials and Methods

### 4.1. Sample Acquisition

Materials and concentrations were chosen to mimic industrial adulteration as closely as possible, with the inclusion of both non-food chemical compounds and amino acids as adulterants in their typical concentration ranges. Whey, pea, beef protein powder, taurine, and glycine were acquired from Scitec Ltd. (Dunakeszi, Hungary). Urea and melamine were provided by Elemental SRL (Bihor, Romania) and Sigma-Aldrich Corporation (Burlington, MA, USA), respectively. Each of the protein powders was artificially adulterated using melamine (M), urea (U), glycine (G), and taurine (T) as adulterants. The nitrogen contents (N) of the adulterants were melamine (66.60% N), urea (46.62% N), glycine (18.65% N), and taurine (11.19% N). Due to the relative difference in nitrogen content among these adulterants, melamine and urea were used in an overall lower concentration than the amino acids (glycine and taurine), which aligns with industrial applications and previous research [1,38,44]. All the powdered samples were prepared to have the same particle size.

### 4.2. Sample Preparation

For each protein powder, a combination pattern was developed to contain single adulterant mixtures (U, G, T, M), dual mixtures (UG, UT, UM, GT, GM, TM), and multiple mixtures (UGT, GTM, UGM, UTM, UGTM). This resulted in a total of 15 different mixture combinations per protein powder, excluding control samples without adulterants. All the mixture combinations were prepared to have the total adulteration level (%*w*/*w*) equivalent to 0.5, 1, 1.5, 2, 2.5, and 3% of melamine in each of the three protein powders. Samples belonging to the same adulteration level had an identical amount of added (external) nitrogen content, resulting in different concentration ranges for each individual adulterant: 0.13–3% for melamine, 0.18–4.29% for urea, 0.45–10.71% for glycine, and 0.74–17.86% for taurine. Triplicates of mixtures were prepared with each weighing 3 g (total mass after adulteration), followed by rigorous homogenization, resulting in 273 samples per protein powder type and 819 samples in total. In addition, pure adulterant samples were also prepared in triplicate. A barcode system was used for easy labeling and identification during scanning with the instruments.

### 4.3. Near-Infrared Spectroscopy Measurements

Three benchtops and one handheld NIR spectrophotometer were used for spectral acquisition. Devices were selected to provide multiple ways of making comparisons, including portability, spectrum-acquisition technologies, and recording ranges. A FOSS NIRSystems 6500 (FOSS NIRSystems, Inc., Silver Spring (Laurel), MD, USA) spectrophotometer with a dispersive grating monochromator was used to acquire spectra in the 400–2498 nm wavelength range at 2 nm intervals. Diffuse reflectance signals were collected by a silicon (Si) detector in the 400–1098 nm range and a lead sulfide (PbS) detector in the 1100–2498 nm range. The MetriNIR (MetriNIR Research, Development, and Service Co., Budapest, Hungary) with a similar grating optical configuration equipped with an indium gallium arsenide (InGaAs) photodetector was used as the second benchtop instrument in the wavelength range of 740–1700 nm and a spectral resolution of 2 nm to acquire diffuse reflectance spectra. A Fourier transform NIR (FT-NIR) spectrometer (MPA, Bruker Optics, Ettlingen, Germany) was used as the third benchtop device to collect diffuse reflectance data in the 4000 to 12,000 cm^−1^ range at 8 cm^−1^ intervals using an integrating sphere and a thermo-regulated so-called extended InGaAs detector. The NIR-S-G1 (InnoSpectra Co., Hsinchu, Taiwan) with a DLP micromirror array and a single InGaAs detector was used as the handheld instrument in the wavelength range of 900–1700 nm and a spectral resolution of 3 nm.

**Figure 8:**
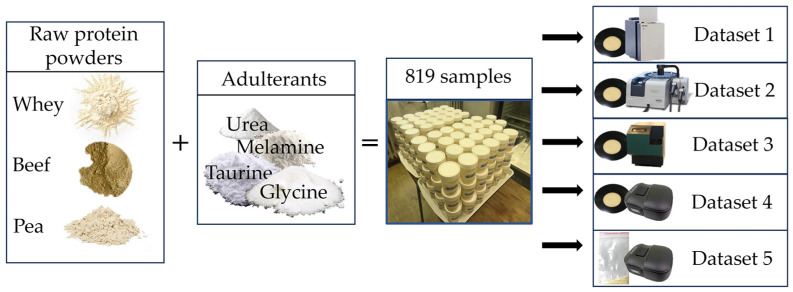
Method of preparing adulterated protein powder samples and spectral datasets with multiple devices.

Samples were measured through an optical glass window cuvette using the NIRSystems 6500, the MetriNIR, NIR-S-G1, and FT-NIR devices. To reduce differences in light scattering, the uniformity of compactness of the powdered samples in the cuvette was achieved by gently tapping the cuvette three times against a laboratory work bench prior to spectral acquisition. Spectral data were also recorded with the handheld device for each sample after transferring them into low-density polyethylene (LDPE) zip-lock bags. This arrangement resulted in a total of five datasets, as summarized in Figure 8.

All spectral acquisitions were performed with 3 consecutive scans at room temperature (23–25 °C). The temperature and humidity of the room were monitored using the Voltcraft DL-121TH multi-data logger to reveal any substantial environmental conditions.

### 4.4. Multivariate Data Analysis of NIRS Spectra

#### 4.4.1. Exploratory Data Analysis

The average raw spectra of each unadulterated (control) protein powder sample set and each pure adulterant were visualized. Also, each protein powder type with the highest level of a given adulterant and their second derivatives were plotted to identify prominent absorbance peaks and assess the need for spectral pretreatments.

Various spectral pretreatment methods were applied in different combinations for further data evaluation to reduce the effect of spectral noise and correct baseline variations, namely Savitzky–Golay (SG) smoothing (2nd order polynomial with varying smoothing points between 5 and 41), standard normal variate (SNV), multiplicative scatter correction (MSC), detrending (deTr), first (FD) and second (SD) derivative [45]. Optimal combinations were chosen for each model built with the aim of reaching the highest predictive accuracy possible.

Principal component analysis (PCA) was used for further data visualization, pattern recognition, and outlier detection [46]. Based on the observations of the raw spectra and PCA, for all further evaluation, each dataset was split into sub-datasets based on protein powder types with restricted wavelength ranges selected for each instrument, focusing on regions that proved to contain more information based on the exploratory evaluation.

#### 4.4.2. Linear Discriminant Analysis

Supervised classification was achieved by performing linear discriminant analysis (LDA). Each sub-dataset was separately optimized by adjusting the number of principal components and applying pretreatments. Models were cross-validated by using a stratified split method based on sample replicates: two-thirds of the dataset for training and one-third for predicting, ensuring that each sample is used in both sets.

Grouping was based on adulteration levels (levels 0–6), visualized by LDA score plots, while correct recognition (calibration) and prediction (cross-validation) percentages were obtained using a confusion matrix. A one-way analysis of variance (ANOVA) was used to determine if there was a significant difference between the classification accuracy of the models built based on different protein types and devices. Homogeneity of variances and normality of residuals were checked using Bartlett’s test and Shapiro-Wilk test, respectively, prior to each ANOVA.

#### 4.4.3. Partial Least Squares Regression

The prediction of adulterant and protein powder concentrations was achieved with partial least squares regression (PLSR). Models were built for each sub-dataset, predicting each adulterant and protein powder content, resulting in a total of 75 models. Models were separately optimized by picking the most suitable pretreatment combination and latent variable number, while the absence of overfitting was verified by observing regression vectors and model metrics [47]. Models were validated with leave-one-replicate-out cross-validation, while the robustness of the best-performing benchtop and handheld datasets was further evaluated with test-set prediction. Test-set prediction was achieved by using two-thirds (two of the replicates) of the dataset for training and one-third (one of the replicates) for predicting.

To determine the minimum adulterant concentration that can be detected and quantified with the PLSR models built, limit of detection (LOD) and limit of quantification (LOQ) values were calculated for each model, following the method described by Allegrini & Olivieri (2014) [48], summarized in the equations below:

(1) $$LOD_{min}=3.3\;{\left[SEN^{-2}\;var\left(x\right)+h_{0min\;}\;SEN^{-2}\;var\left(x\right)+h_{0min\;}var\left(y_{cal}\right)\right]}^{1/2}$$

(2) $$LOD_{max}=3.3\;{\left[SEN^{-2}\;var\left(x\right)+h_{0max\;}\;SEN^{-2}\;var\left(x\right)+h_{0max\;}var\left(y_{cal}\right)\right]}^{1/2}$$

where SEN means the sensitivity (inverse of the length of the regression coefficient) and var(x) is the variance in instrument signals. h_0min/_ h_0max_ is the minimum/maximum distance between a hyperplane for the calibration set, indicating the center of a normalized calibration score space and the scores of the samples for which the analyte of interest is absent. var(y_cal_) is the variance in the calibration (adulterant) concentrations [48]. The limit of detection is the analyte level, which, with a sufficiently high probability, will lead to a correct positive detection decision [49]. The lower and upper limits of the LOD interval (LOD_min_ and LOD_max_) correspond to the calibration samples with the lowest and largest extrapolated leverages to zero analyte concentration. The LOQ interval was obtained by multiplying the LOD values with a factor value of three [50].

The following metrics were used to evaluate PLSR models: root mean square error of calibration (RMSEC), root mean square error of cross-validation (RMSECV), root mean square error of test-set prediction (RMSEP), determination coefficient of calibration (R^2^C), determination coefficient of cross-validation (R^2^CV), determination coefficient of test-set prediction (R^2^P), limit of detection minimum value (LODmin), limit of detection maximum value (LODmax), limit of quantification minimum value (LOQmin), and limit of quantification maximum value (LOQmax).

Data evaluation and visualization were achieved with R-project (v. 4.3.0, 2023, The R Foundation for Statistical Computing, Vienna, Austria; using R package: aquap2 [36]), MATLAB (Version: 23.2.0.2428915 (R2023b), The MathWorks Inc. (2023), Natick, MA, USA), and Microsoft 365 Excel (Microsoft Corporation, Redmond, WA, USA).

## Figures and Tables

**Table 1:** PLSR results based on each sub-dataset to predict protein powder concentration.

	Whey					Beef					Pea				
Instrument	NIRS6500	MPA	MetriNIR	NIR-S-G1	NIR-S-G1 (Bag)	NIRS6500	MPA	MetriNIR	NIR-S-G1	NIR-S-G1 (Bag)	NIRS6500	MPA	MetriNIR	NIR-S-G1	NIR-S-G1 (Bag)
Range (wl)	1100–2200	1400–2200	950–1650	950–1650	950–1650	1100–2200	1400–2200	950–1650	950–1650	950–1650	1100–2200	1400–2200	950–1650	950–1650	950–1650
Pre-treat.	SG 15 + SNV	SG 21 + SC	SG 35 + SNV	SG 21 + SNV	SG 25 + SNV	SG 21 + SC	SG 41 + SNV	SG 35 + SNV	SG 11 + SNV	SG 21 + SNV	SG 5 + SNV	SG 21 + SNV	SG 35 + SNV	SG 19 + SNV	SG 27 + SNV
LV	12	4	6	5	7	12	11	5	7	6	6	10	6	10	7
RMSEC	0.65	1.05	1.09	1.52	1.22	0.71	0.86	0.97	1.21	1.24	0.76	0.74	0.90	1.16	1.22
R^2^C	0.97	0.92	0.91	0.82	0.89	0.96	0.94	0.93	0.89	0.88	0.96	0.96	0.94	0.90	0.89
RMSECV	0.71	1.07	1.14	1.58	1.26	0.79	0.91	1.01	1.25	1.30	0.81	0.79	0.94	1.22	1.26
R^2^CV	0.96	0.91	0.90	0.81	0.88	0.95	0.94	0.92	0.88	0.87	0.95	0.95	0.93	0.89	0.88

**Table 2:** PLSR results based on each sub-dataset to predict urea concentration.

	Whey					Beef					Pea				
Instrument	NIRS6500	MPA	MetriNIR	NIR-S-G1	NIR-S-G1 (Bag)	NIRS6500	MPA	MetriNIR	NIR-S-G1	NIR-S-G1 (Bag)	NIRS6500	MPA	MetriNIR	NIR-S-G1	NIR-S-G1 (Bag)
Range (wl)	1100–2200	1400–2200	950–1650	950–1650	950–1650	1100–2200	1400–2200	950–1650	950–1650	950–1650	1100–2200	1400–2200	950–1650	950–1650	950–1650
Pre-treat.	SG 9 + SNV	SG 21 + 2der	SG 9 + SNV	SG 15 + SNV	SG 11 + SNV	SNV	SG 21 + SC	SG 21 + SNV	SG 11 + SNV	SG 21 + SNV	SNV	SG 31 + SC	SG 5 + SNV	SG 11 + SNV	SG 15 + SNV
LV	4	3	5	8	7	5	6	4	10	8	5	12	4	10	7
RMSEC	0.19	0.20	0.20	0.27	0.23	0.18	0.17	0.21	0.24	0.25	0.19	0.15	0.20	0.23	0.24
C	0.95	0.95	0.94	0.90	0.93	0.96	0.96	0.94	0.92	0.91	0.95	0.97	0.94	0.93	0.92
RMSECV	0.19	0.21	0.21	0.28	0.24	0.18	0.18	0.23	0.25	0.26	0.19	0.16	0.21	0.24	0.25
R^2^CV	0.95	0.94	0.94	0.89	0.92	0.95	0.95	0.93	0.91	0.91	0.95	0.96	0.94	0.92	0.91
LODmin	0.08	0.14	0.09	0.27	0.25	0.04	0.06	0.20	0.26	0.46	0.04	0.08	0.16	0.26	0.30
LODmax	0.15	0.20	0.18	0.50	0.40	0.13	0.15	0.24	0.47	0.53	0.13	0.17	0.21	0.37	0.35
LOQmin	0.24	0.43	0.26	0.82	0.75	0.13	0.17	0.60	0.77	1.37	0.13	0.25	0.48	0.78	0.90
LOQmax	0.45	0.60	0.55	1.51	1.19	0.39	0.44	0.73	1.42	1.60	0.40	0.52	0.63	1.11	1.06

**Table 3:** PLSR results based on each sub-dataset to predict melamine concentration.

	Whey					Beef					Pea				
Instrument	NIRS6500	MPA	MetriNIR	NIR-S-G1	NIR-S-G1 (Bag)	NIRS6500	MPA	MetriNIR	NIR-S-G1	NIR-S-G1 (Bag)	NIRS6500	MPA	MetriNIR	NIR-S-G1	NIR-S-G1 (Bag)
Range (wl)	1100–2200	1400–2200	950–1650	950–1650	950–1650	1100–2200	1400–2200	950–1650	950–1650	950–1650	1100–2200	1400–2200	950–1650	950–1650	950–1650
Pre-treat.	SNV	SG 25 + SNV	SG 21 + SNV	SG 9 + SNV	SG 15 + SNV	SC	SG 21 + SNV	SG 21 + SNV	SG 5 + deTr	SG 11 + SNV	SNV	SG 21 + SNV	SG 21 + SNV	SG 15 + SC	SG 19 + SNV
LV	6	6	6	7	7	6	6	6	6	7	4	6	6	10	9
RMSEC	0.14	0.16	0.15	0.24	0.24	0.16	0.16	0.15	0.23	0.26	0.17	0.16	0.14	0.24	0.22
R^2^C	0.94	0.93	0.94	0.83	0.83	0.93	0.92	0.94	0.85	0.81	0.91	0.93	0.94	0.84	0.86
RMSECV	0.15	0.16	0.16	0.25	0.26	0.17	0.17	0.16	0.23	0.27	0.18	0.17	0.15	0.24	0.23
R^2^CV	0.94	0.92	0.93	0.82	0.81	0.92	0.92	0.93	0.84	0.80	0.91	0.92	0.94	0.83	0.84
LODmin	0.03	0.04	0.07	0.33	0.25	0.03	0.06	0.10	0.33	0.31	0.07	0.05	0.08	0.26	0.24
LODmax	0.13	0.21	0.21	0.55	0.40	0.12	0.15	0.18	0.55	0.45	0.15	0.15	0.17	0.37	0.29
LOQmin	0.10	0.12	0.22	1.00	0.75	0.10	0.17	0.29	1.00	0.94	0.20	0.16	0.24	0.78	0.71
LOQmax	0.40	0.64	0.64	1.66	1.19	0.37	0.44	0.55	1.66	1.35	0.46	0.46	0.52	1.11	0.87

**Table 4:** PLSR results based on each sub-dataset to predict taurine concentration.

	Whey					Beef					Pea				
Instrument	NIRS6500	MPA	MetriNIR	NIR-S-G1	NIR-S-G1 (Bag)	NIRS6500	MPA	MetriNIR	NIR-S-G1	NIR-S-G1 (Bag)	NIRS6500	MPA	MetriNIR	NIR-S-G1	NIR-S-G1 (Bag)
Range (wl)	1100–2200	1400–2200	950–1650	950–1650	950–1650	1100–2200	1400–2200	950–1650	950–1650	950–1650	1100–2200	1400–2200	950–1650	950–1650	950–1650
Pre-treat.	SG 21 + SC	SG 31 + SNV	SG 21 + SNV	SG 27 + SNV	SG 15 + SNV	SC	SG 21 + SNV	SG 35 + SNV	SG 11 + SNV	SG 21 + SNV	SNV	SG 21 + SNV	SG 35 + SNV	SG 21 + SNV	SG 15 + SNV
LV	11	11	6	10	8	4	6	6	8	10	4	6	6	9	8
RMSEC	0.35	0.71	0.88	1.26	1.24	0.51	0.78	0.82	1.18	1.18	0.48	0.74	0.77	1.14	1.24
R^2^C	0.97	0.96	0.94	0.87	0.88	0.94	0.95	0.95	0.89	0.89	0.95	0.96	0.95	0.90	0.88
RMSECV	0.40	0.80	0.93	1.32	1.29	0.53	0.84	0.87	1.23	1.22	0.50	0.79	0.82	1.19	1.29
R^2^CV	0.96	0.95	0.93	0.86	0.87	0.94	0.94	0.94	0.88	0.88	0.94	0.95	0.95	0.89	0.87
LODmin	0.06	0.03	0.07	0.23	0.24	0.08	0.06	0.10	0.23	0.30	0.07	0.06	0.08	0.30	0.31
LODmax	0.18	0.31	0.25	0.45	0.39	0.16	0.15	0.18	0.45	0.38	0.15	0.15	0.18	0.40	0.36
LOQmin	0.18	0.10	0.22	0.70	0.71	0.23	0.18	0.29	0.70	0.91	0.20	0.17	0.24	0.89	0.93
LOQmax	0.54	0.94	0.75	1.34	1.17	0.47	0.45	0.55	1.34	1.15	0.46	0.46	0.54	1.20	1.08

**Table 5:** PLSR results based on each sub-dataset to predict glycine concentration.

	Whey					Beef					Pea				
Instrument	NIRS6500	MPA	MetriNIR	NIR-S-G1	NIR-S-G1 (Bag)	NIRS6500	MPA	MetriNIR	NIR-S-G1	NIR-S-G1 (Bag)	NIRS6500	MPA	MetriNIR	NIR-S-G1	NIR-S-G1 (Bag)
Range (wl)	1100–2200	1400–2200	950–1650	950–1650	950–1650	1100–2200	1400–2200	950–1650	950–1650	950–1650	1100–2200	1400–2200	950–1650	950–1650	950–1650
Pre-treat.	SG 21 + SNV	SG 21 + SNV	SG 21	SG 25	SG 21 + 2der	SG 15 + SNV	SG 21 + SNV	SG 25	SG 11 + deTr	SG 21 + SNV	SNV	SG 31 + SNV	SG 31	SG 21 + SNV	SG 21 + SNV
LV	9	5	8	12	9	8	4	8	7	7	6	4	6	10	8
RMSEC	0.63	0.50	0.63	0.90	0.81	0.67	0.65	0.61	0.71	1.01	0.69	0.53	0.50	0.83	0.96
R^2^C	0.97	0.94	0.91	0.82	0.86	0.96	0.91	0.92	0.89	0.77	0.96	0.94	0.94	0.85	0.79
RMSECV	0.68	0.52	0.67	0.95	0.84	0.73	0.69	0.65	0.74	1.03	0.74	0.55	0.53	0.87	1.00
R^2^CV	0.96	0.94	0.90	0.80	0.84	0.96	0.89	0.90	0.88	0.76	0.96	0.93	0.94	0.83	0.78
LODmin	0.05	0.09	0.06	0.20	0.12	0.04	0.08	0.09	0.32	0.31	0.04	0.08	0.12	0.32	0.33
LODmax	0.20	0.17	0.26	0.54	0.31	0.14	0.18	0.22	0.42	0.45	0.14	0.17	0.22	0.44	0.38
LOQmin	0.14	0.26	0.18	0.60	0.36	0.11	0.23	0.28	0.96	0.94	0.13	0.23	0.37	0.96	0.99
LOQmax	0.59	0.51	0.77	1.62	0.93	0.41	0.53	0.67	1.25	1.35	0.43	0.50	0.65	1.31	1.14

## Data Availability

Data will be made available upon request.
